# Unlocking the Potential of Spent Mushroom Substrate (SMS) for Enhanced Agricultural Sustainability: From Environmental Benefits to Poultry Nutrition

**DOI:** 10.3390/life13101948

**Published:** 2023-09-22

**Authors:** Filipa Baptista, Mariana Almeida, Jéssica Paié-Ribeiro, Ana Novo Barros, Miguel Rodrigues

**Affiliations:** 1 Centre for the Research and Technology of Agro-Environmental and Biological Sciences, CITAB, University de Trás-os-Montes e Alto Douro, UTAD, 5000-801 Vila Real, Portugal; abarros@utad.pt (A.N.B.); mrodrigu@utad.pt (M.R.); 2 Veterinary and Animal Research Centre (CECAV), Associate Laboratory of Animal and Veterinary Sciences (AL4AnimalS), University of Trás-os-Montes e Alto Douro, Quinta de Prados, 5000-801 Vila Real, Portugal; mdantas@utad.pt (M.A.); jessicapaie@utad.pt (J.P.-R.)

**Keywords:** spent mushroom substrate, antioxidant, antibiotic, fertilizer

## Abstract

In this comprehensive review, we delve into the myriad applications of spent mushroom substrate (SMS) in agricultural contexts, with a particular emphasis on its role in fostering sustainable poultry production. Our examination spans three key domains: the use of SMS in fertilizers, its impact on environmental factors and gas emissions, and its contribution to poultry nutrition. This review synthesizes findings from multiple studies that underscore the potential of composted SMS as a viable alternative to conventional inorganic fertilizers, effectively meeting crop nutrient needs while mitigating groundwater contamination risks. Moreover, we highlight the substantial environmental advantages associated with the utilization of SMS and poultry waste, including reductions in greenhouse gas emissions and the promotion of sustainable waste management practices. Additionally, we explore the promising outcomes of integrating SMS into animal feed formulations, which have demonstrated significant enhancements in livestock growth performance and overall health. In sum, this review underscores the versatility and untapped potential of SMS as a valuable agricultural resource, with a particular focus on its role in advancing sustainable practices, optimizing nutrient management, and harnessing the value of organic waste materials, especially in the context of poultry production.

## 1. Introduction

The taxonomy of mushrooms currently encompasses a vast array of fungi, comprising approximately 12,000 species, among which more than 2000 species are distinguished as edible and possess therapeutic attributes, resulting in their widespread consumption [1,2,3]. In 2018, the global mushroom market achieved a notable volume of around 13 million tons, with projections indicating a substantial growth to 21 million tons by 2026 [4,5,6,7].

As the mushroom industry advances, it yields a consequential by-product known as spent mushroom substrate (SMS). Comprising residual fungal mycelium, lignocellulosic biomass, and enzymes, SMS has garnered significant attention as a substantial waste product [7,8,9]. The composition of raw SMS can vary, with contents of up to 48.7% cellulose, 34% hemicellulose, and 39.8% lignin, contingent upon the source of the mushroom cultivation medium. SMS also serves as a source of essential vitamins and minerals, including iron, magnesium, zinc, and calcium. Moreover, it is remarkably rich in bioactive compounds, encompassing polysaccharides, polypeptides, and phenolics. SMS additionally harbors valuable enzymes; research has indicated that SMS from Lentinula edodes, Agaricus bisporus, and Pleurotus eryngii possesses enzymes such as β-glucanase, xylanase, laccase, and phytase [7].

**Figure 1:**
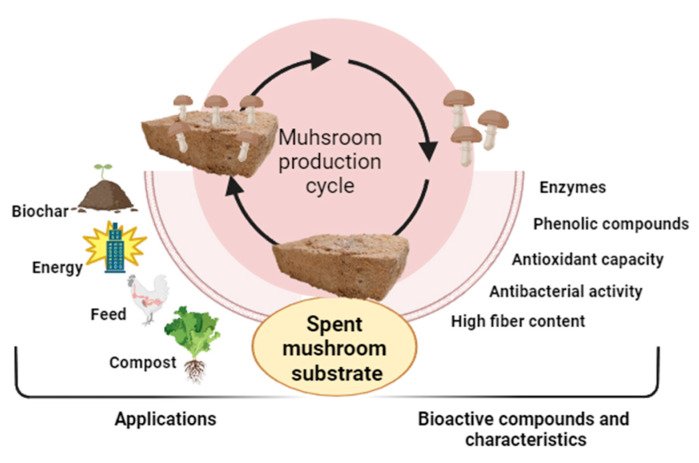
Mushroom production cycle and spent mushroom substrate bioactive compounds and main applications.

However, as the edible mushroom sector thrives, SMS accumulates at a remarkable rate, approximately 5 kg for every kilogram of freshly harvested mushrooms, culminating in a staggering 60 million tons over a decade [5,7]. Regrettably, SMS frequently encounters disposal as agricultural waste, incurring substantial costs ranging from 10 to 50 EUR/ton in Europe, thus accumulating a considerable financial burden that could potentially surpass EUR 150 million annually for the industry [8]. The absence of sustainable disposal strategies emerges as a significant hindrance to the continued expansion of the mushroom industry [10]. Arguably, as illustrated in Figure 1, SMS enzyme extraction and bioactive compound potential emerge as promising avenues [11,12]. The incorporation of SMS as animal feed has been practiced for several decades, with *P. ostreatus* and *L. edodes* being used in ruminants [13]. Beyond ruminants, SMS integration into livestock, poultry, and fish diets enhances microbial balance and promotes growth [7]. SMS’s potential as a feed source is evident in various species [14], including insects (Beetle: *Protaetia Brevitarsis*; Mealworm: *Tenebrio molitor*) [15,16], rabbits (Cuban Brown: *Oryctolagus cuniculus*) [17], pigs (*Sus scrofa domesticus*) [18], and ruminants (Sika deer: *Cervus nippon*, Dairy cows: *Bos taurus coreanae*, *Bos taurus*) [19,20,21]. Similarly, to mushrooms’ fruiting bodies, whose biological activity is far more studied, SMS can exhibit nutraceutical properties worth studying, such as antioxidant capacity [22,23,24], antibacterial and antifungal activity [25,26,27], antiaging activity [28], and other health benefits [29]. 

In the realm of agriculture, spent mushroom substrate (SMS) emerges as a compelling sustainable alternative to conventional chemical fertilizers and soil amendments, offering solutions to both energy consumption and environmental concerns [7,30]. Remarkably, even after undergoing multiple cycles of mushroom production, SMS retains its robust nutrient content and organic matter, rendering it an ideal choice for biofertilization and soil enhancement [10,31]. The distinctive qualities of SMS, including excellent air permeability, water and nutrient retention capabilities, and a loose texture, foster an improved ecological environment for soil microorganisms and enhance the physical structure of the soil, thereby minimizing plant stress and bolstering nutrient bioavailability [7,31]. Furthermore, SMS exhibits the capacity to fine-tune soil pH levels and mitigate soil contamination by various pollutants such as heavy metals, pesticides, and polycyclic aromatic hydrocarbons [31,32].

Beyond its applications in agriculture, SMS holds substantial promise in the realm of renewable energy production from agro-industrial biomass [30,33]. It seamlessly integrates into various bioenergy production methods, bolstering the generation of biomass fuels, biofuels, bioethanol, and biogas [30,34]. Co-digestion of SMS with other lignocellulosic biomass sources has displayed significant potential, particularly in the enhancement of methane and hydrogen production [30,34]. This collaborative approach not only advances renewable energy objectives but also contributes to cost reduction in operational processes [35]. Expanding its role, SMS emerges as a bioremediation contender, targeting environmental contamination through living organisms [36,37]. SMS from various mushroom species effectively removes pollutants like H_2_S, volatile compounds, and heavy metals, even degrading harmful compounds like polycyclic aromatic hydrocarbons and pesticides. This underscores SMS’s potential for remediating polluted soils and waters [32,36,38].

The goal of this review article is to systematically examine and analyze existing research articles that demonstrate the utilization of spent mushroom substrate in poultry production or in conjunction with poultry production by-products. Specifically, we aim to evaluate how SMS serves as an interesting by-product with diverse potential applications. Through this comprehensive review, we seek to contribute to a deeper understanding of the multifaceted role of SMS in sustainable agriculture, waste management, and various related domains.

## 2. Methodology

This study adopted an applied approach, in line with Hiebl’s framework [39], with the primary objective of scrutinizing the methods employed for sample selection in systematic literature reviews within the field of management research. The study’s overarching aims were exploratory and descriptive in nature, facilitating a comprehensive evaluation of the data and the acquisition of interpretative insights. The technical procedures employed in this research closely adhered to the systematic literature review framework, following the well-established steps delineated by Cronin et al. [40]. These steps encompassed formulating the research question (I), identifying pertinent databases (II), devising search strategies, selecting, and accessing pertinent literature (III), evaluating the quality of the studies included (IV), and ultimately, conducting an in-depth analysis, synthesis, and dissemination of the research findings (V).

### 2.1. Formulate the Research Question (I) 

Research Question: What is the scientific research scenario on using spent mushroom substrate in poultry production?

### 2.2. Searched Databases and the Search Strategies (II)

Conducting a systematic review depends on the scope and quality of the included studies (15). Thus, to recover scientific articles of proven quality, we sought to use only electronic bases that retrieve journals that perform peer reviews of a manuscript, which were national and international. The bases used were Web of Science, Scopus, PubMed, National Agricultural Library (USDA), CABI (ASD), ScienceDirect, and Scielo. According to Levy and Ellis (16), this step is called entry. Next, each database’s descriptors and Booleans used as search guidance were determined. The search method varied several times among databases because they have some peculiarities. Boolean operators were for establishing selection criteria and thus retrieving the maximum number of papers related to the theme. All bases were last consulted on the 16th of May. The descriptors used as search guidance in each database were determined. Keywords were established according to selection criteria and thus retrieving the maximum number of papers related to the theme. When searching, the AND operator was inserted between two words to obtain articles that contain both (e.g., spent mushroom substrate AND poultry). The operator OR was used to extend the search for articles containing one word or the other (e.g., poultry OR broiler). Keywords between quotation marks were used for refining the search. Therefore, articles were retrieved only if the words appeared together. In all databases, no time/date limit was imposed. After obtaining the results, only scientific articles were selected, excluding books and book chapters, review articles, conference papers, notes, letters, erratum, and editorials. 

#### 2.2.1. Scopus, PubMed, Web of Science, CABI and Scielo

The search in the Scopus, PubMed, Web of Science, ScienceDirect, CABI, and Scielo databases was similar. The following keywords and combinations were used through Booleans to refine the search and exclude irrelevant articles. In the Scopus database, the search was restricted to the article title, abstract, and keywords/topics (searching for words contained only in the article title, abstract, and/or keywords). In contrast, in the rest of the databases, the search was not restricted (All fields) to the following combination: “spent mushroom substrate AND broiler” OR “spent mushroom substrate AND chicken” OR “spent mushroom substrate AND poultry” OR “spent mushroom substrate AND laying hen” OR “spent mushroom AND broiler” OR “spent mushroom AND chicken” OR “spent mushroom AND poultry” OR “spent mushroom AND laying hen”. After obtaining the results, only scientific articles were selected, excluding books and book chapters, review articles, conference papers, notes, letters, erratum, and editorials. 

#### 2.2.2. ScienceDirect and National Agricultural Library

In ScienceDirect and National Agricultural Library (NAL), the same keywords were searched separately, with the first search consisting of: “spent mushroom substrate” AND “broiler”, “spent mushroom substrate” AND “chicken”, “spent mushroom substrate” AND “poultry”, “spent mushroom substrate” AND “laying hen”; and the second search consisted on: “spent mushroom” AND “broiler”, “spent mushroom” AND “chicken”, “spent mushroom” AND “poultry”, “spent mushroom” AND “laying hen”. Duplicates in the same database were discarded. The search was not restricted to any fields in the National Agricultural Library database. The search in the ScienceDirect database was restricted to “abstract”, “title” and/or “keywords”.

### 2.3. Inclusion and Exclusion Criteria (III) 

The articles retrieved from databases were selected according to the following criteria: Only studies where spent mushroom substrate was used in poultry production or combined with poultry production by-products/waste were selected. The focus should be on using spent mushroom substrate not only directly on poultry production but also with poultry production by-products or waste; studies that involved both poultry or its by-products and SMS not combined were excluded. They should obligatorily present a relation between the two. After being selected according to the mentioned criteria, the articles were grouped into different knowledge fields: “Fertilizers”, “Environment and gas emissions” and “Animal nutrition”.

### 2.4. Critical Analysis of the Selected Studies (IV) 

The articles chosen in the previous step were read in full to extract relevant aspects of the objectives, methodology, results, and conclusions. This analysis also considered the quality of the studies, which should have a detailed description of the methodology and conclusive results with a thorough discussion.

### 2.5. Summary of the Results (V) 

The presentation of the results focused on describing the main characteristics of the studies, highlighting the use of spent mushroom substrate in the poultry production industry.

## 3. Results

### Overview of the Selected Research 

**Figure 2:**
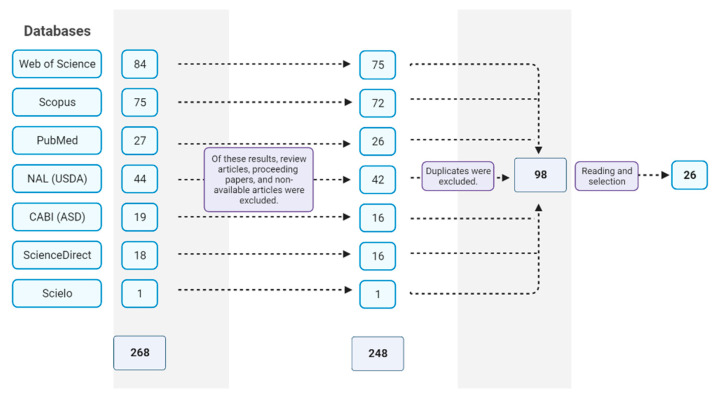
PRISMA flow diagram (animal study). Summary of the literature search, screening, and selection of potential studies. The PRISMA flow diagram shows the literature search in the different electronic databases, Web of Science, Scopus, PubMed, National Agriculture Library, CABI, ScienceDirect, and Scielo, followed by processes of screening and inclusion of eligible studies for the systematic review. Source: research data.

A flow diagram for the literature search summary, screening, and selection of potential studies is shown in Figure 2. After the search using the keywords and Booleans, 268 papers were retrieved. Subsequently, when we used the exclusion criteria of the databases, the studies were reduced by 26.6%, totaling 98 scientific articles, from which 26 were selected (Figure 2).

**Figure 3:**
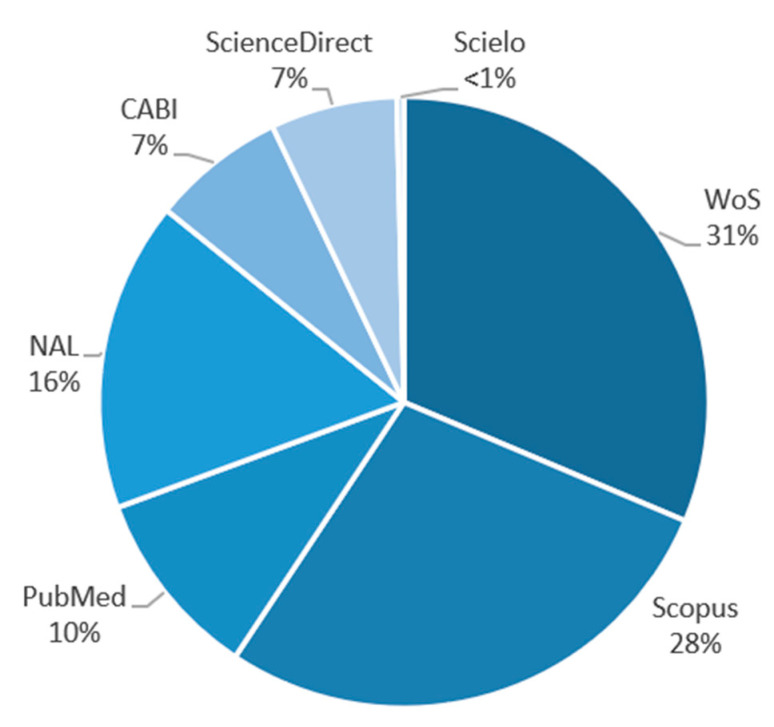
The proportion of articles retrieved from each database. Source: Research data.

**Figure 4:**
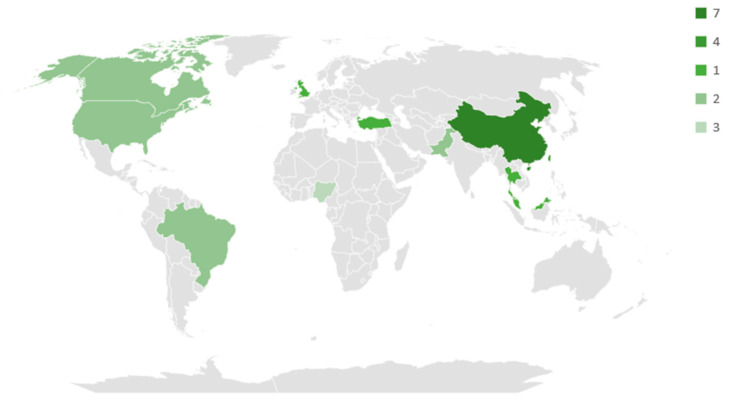
Geographical distribution of the studies. Source: Research data.

Due to its extensive databases, Web of Science (WoS), Scopus, and National Agricultural Library (NAL) databases, reckoning more than 75%, took up most studies. Nevertheless, PubMed, ScienceDirect, and CABI databases retrieved about 24%, a reasonable number of articles to evaluate. However, only one study was retrieved from Scielo (>1%) (Figure 3). As for the geographical location, most of the work was concentrated in Asia (57.69%), followed by North and South America combined (23.08%). A reduced amount of research in recent years has been developed in Africa (11.54%) and Europe (7.69%) (Figure 3). Figure 4 illustrates the categorization of all selected research. Most of the published papers from the last five years focused on environment and gas emissions (53.33%), with a considerable number of selected works in agriculture and fertilizers (33.33%). Fewer studies were found related to animal nutrition within the last five years (13.33%).

The selected research could be divided into three fields of study: fertilizers, environment and gas emissions, and animal nutrition. From the overall studies, spent mushroom substrate as a replacement for fertilizers was the field of study with more articles (50%), while “Animal nutrition” (19.23%) and “Renewable energy and gas emissions” (30.77%) are areas in potential development. 

## 4. Discussion

### 4.1. Characterization of the Selected Research 

#### 4.1.1. Alternatives to Conventional Fertilizers

**Figure 5:**
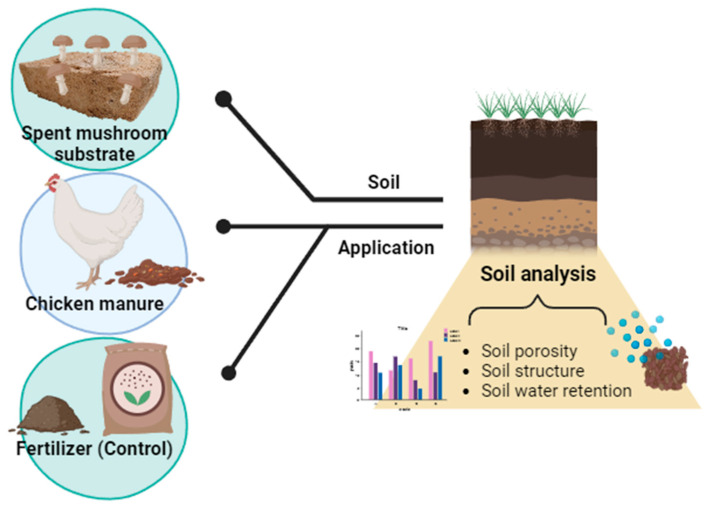
Effects of application of recycled chicken manure and spent mushroom substrate on organic matter, acidity, and hydraulic properties of sandy soils [42].

Soil organic matter plays a central role in driving soil processes and functions and is a key resource base for agriculture. As demonstrated in Figure 5, it contributes to an improvement in soil structure, water retention, and quality. The incorporation of residual spent mushroom substrate that remains after the mushroom harvesting process, along with the poultry manure obtained from waste disposal has gained substantial endorsement for its advantageous role in agricultural recycling. This practice promotes the establishment of entirely natural nutrient and organic carbon cycles, aligning with the pursuit of enduring sustainability. Both organic materials hold significant value as constituents of composts. Their inherent alkaline characteristics contribute to the amelioration of soil acidity while simultaneously augmenting the accessibility of nutrients [41]. Furthermore, the utilization of poultry manure and litter extends to serving as a source of chemical energy for the generation of electricity and heat, whereas SMS finds application as a substrate for other mushroom-forming fungi and becomes integral to the production of biofuels and enzymes [42].

As previously stated, the field of study primarily revolved around fertilizers, particularly the co-composting of spent mushroom substrate (SMS) and poultry manure. The central objective was to explore their potential as substitutes for conventional inorganic fertilizers. Leading this investigation, Maynard [43,44] was one of the first to study this matter in 1993 and 1994. The core focus was to determine whether composted animal manures, specifically SMS and poultry manure, could effectively serve as a nutrient source for intensive vegetable production systems, while also assessing their potential impact on groundwater quality. Maynard’s pioneering studies unveiled intriguing insights. He ventured into a comprehensive analysis of compost compositions. The SMS compost consisted of horse manure and bedding amended with cottonseed, gypsum, chicken manure (CM), and cocoa bean shells. In contrast, the chicken manure compost consisted of chicken manure (43%), horse manure, SMS, and sawdust. Following yearly applications of these composts as the sole source of nutrients, yields of seven crops from these amended plots were compared to yields from control plots fertilized with NPK fertilizer. The CM compost-amended plots had similar or better results compared to the fertilizer used as a control in all three years, with one exception [43]. Nitrate concentrations in ground water from beneath all compost-amended plots were assessed and the results showed they remained below 10 ppm during the study, while concentrations beneath the fertilized control climbed to 14.7 ppm. 

Additionally, studies by Zailani and Hamid [45] and Udom et al. [46] examined the effects of SMS and poultry waste on composting and soil improvement. Zailani and Hamid highlighted the role of microbial inoculums in enhancing the composting process of SMS, including poultry waste, while Udom et al. demonstrated that the application of poultry manure and SMS improved soil structure and water infiltration, particularly beneficial for poultry farming. The present findings indicate that the composts under investigation may serve as a viable substitute for inorganic fertilizers in intensive cropping systems, fulfilling a significant portion of the nutrient requirements. However, caution must be exercised with respect to the cumulative impact on soil quality resulting from annual additions, as well as the potential for nitrate contamination of groundwater.

A study was conducted to assess the physical and chemical characteristics of substrates comprising spent mushroom substrate and poultry manure compost, as well as cattle manure in varying proportions, and their impact on nutrient uptake and growth responses of honeydew melon seedlings. The study aimed to determine the optimal mixture for honeydew seedling production in organic agriculture. The substrates containing SMS and manure exhibited physical properties within the desirable range, but the use of a high proportion of manure (80%) resulted in reduced germination and survival rates of honeydew melon seedlings [47]. A similar study evaluated the effects of spent mushroom substrate and poultry manure compost on honeydew melons’ growth, yield, and fruit quality to determine the most suitable SMS rate for honeydew melon production. The results indicated that increasing organic materials increased honeydew melon’s growth, yield, and quality due to enhancing pH, organic matter, and nutrient concentrations in the soil [48].

Spent mushroom substrate (SMS) has emerged as a focal point in agricultural practices, particularly in the realms of waste management and organic fertilization. SMS boasts a rich composition of organic and mineral matter, with a noteworthy emphasis on essential elements like nitrogen, phosphorus, and potassium [49]. Moreover, its abundance in organic material renders it a highly sought-after soil amendment. SMS lends itself effectively to composting when combined with other organic waste materials, including chicken feathers and manure. Ali et al. [49] conducted an enlightening investigation into the response of various onion cultivars to different organic manures, encompassing farmyard manure, poultry manure, and spent mushroom compost. Their findings underscored the significant enhancements in growth and yield achieved through SMS application, ultimately concluding that SMS holds great promise as a potent organic manure option for bolstering onion production.

Similarly, Hussain et al. [50] delved into the effects of organic and inorganic regimes on cauliflower growth, production, and quality characteristics. Their results illuminated the substantial improvements realized across these parameters with SMS application, highlighting its efficacy as an organic fertilizer for promoting robust cauliflower growth and enhancing its nutritional quality, particularly when complemented by the inclusion of poultry waste as a nutrient source. In their research, Rao et al. [51] employed pelleted organo-mineral fertilizers derived from composted pig slurry solids, animal waste, and spent mushroom substrate for enhancing amenity grasslands, which they aptly described as “golf courses.” The study’s outcomes revealed that these pelleted fertilizers exerted a positive influence on grass growth, shedding light on the promising role of SMS in sustainable nutrient management and its potential to bolster the productivity of grasslands.

Shifting our focus to the dynamics of soil nutrients, Li et al. [52] conducted an insightful in situ field incubation study in apple orchard soil. Their investigation centered on the impact of composted manure and chemical fertilizers, including SMS derived from poultry waste. The results unveiled that the combined application of composted manure and chemical fertilizers, featuring SMS, significantly influenced soil nutrient availability. These findings strongly suggest that the inclusion of SMS in composted manure can enhance nutrient cycling and promote sustainable orchard management.

Exploring innovative uses of waste materials, two studies ventured into the potential of composted media crafted from waxed corrugated cardboard as a soil amendment and growing medium for container-grown woody ornamentals. These investigations showcased that these composted media, which incorporated poultry waste-derived SMS, exhibited favorable physical and chemical characteristics, rendering them suitable for stimulating plant growth and serving as eco-friendly alternatives to conventional growing media [53,54].

Collectively, the studies presented herein underscore the substantial potential of integrating spent mushroom substrate (SMS) as a valuable asset in sustainable agriculture. Through inquiries into its integration into composting processes and its impact on diverse crops, these studies illuminate SMS’s capacity to enhance soil quality, stimulate plant growth, and elevate yields. These highlighted benefits, combined with its rich organic and mineral composition, position SMS as a promising organic fertilizer and waste management solution. This opens up exciting avenues for its incorporation into a wide spectrum of agricultural practices, fostering both enhanced productivity and environmental responsibility.

Shifting our focus to co-composting practices, Xie et al. [55] delved into the biochemical and microbiological properties during the co-composting of spent mushroom substrate and chicken feathers. Their study effectively demonstrated the prowess of the co-composting process in breaking down waste material, proving that SMS is a feasible composting feedstock due to its abundant fungus. These findings underscore the considerable potential of co-composting SMS and poultry waste as a viable waste management strategy, yielding nutrient-rich compost while addressing environmental concerns. Kulcu et al. [56] delved into the intricate process of co-composting, involving spent mushroom substrate, carnation wastes, poultry, and cattle manure. Their research yielded compelling evidence that the co-composting of these materials resulted in the creation of stable compost possessing enhanced physical and chemical characteristics. This discovery underscores the practicality of co-composting as a waste management strategy, simultaneously generating high-quality compost tailor-made for agricultural applications.

In a thorough and comprehensive assessment, Jia et al. [57] embarked on an exploration of the co-composting realm, specifically focusing on the synergy between spent mushroom substrate and poultry manure, with the addition of garden waste. Their meticulous study meticulously scrutinized the physicochemical attributes, humification process, and spectral characteristics of dissolved organic matter throughout the co-composting journey. The findings unveiled that the introduction of garden waste significantly bolstered the composting process, culminating in compost possessing favorable attributes and heightened organic matter stability. These collective findings highlight co-composting’s potential, serving both as an effective waste management strategy and a means of generating nutrient-rich compost ideally suited for sustainable agricultural practices.

Lastly, with a focus on the microbial intricacies inherent in composting, Lin et al. [58] undertook a comprehensive investigation into the dynamic shifts within the microbial community during the large-scale co-composting of swine and poultry manure with spent mushroom substrate. Their results illuminated a dynamic transformation in the composition of the microbial community, accompanied by an increase in microbial diversity throughout the co-composting process. This dynamic shift in microbial composition can be attributed, in part, to the alterations in physiochemical properties resulting from the addition of manure. Such insights into microbial dynamics are pivotal for comprehending the underlying mechanisms and ensuring the production of high-quality compost. These findings highlight the importance of bacterial and fungal dynamics when considering an effective waste management strategy.

#### 4.1.2. Renewable Energy and Gas Emissions

**Figure 6:**
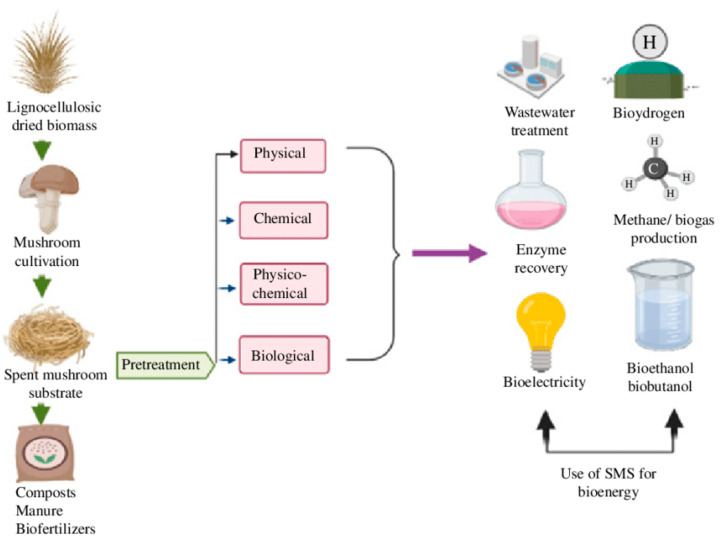
Sustainable utilization of spent mushroom substrate (SMS) for energy production.

Spent mushroom substrate (SMS) and poultry waste stand as formidable allies in the battle against greenhouse gas emissions and environmental degradation. These organic resources hold immense promise, finding effective utilization avenues in composting processes, biogas production, and precision nutrient management strategies [59,60,61]. By redirecting these valuable waste streams away from landfills and harnessing their inherent attributes, we can take substantial strides towards sustainable waste management, climate change mitigation, and the promotion of a more robust ecosystem. The utilization of SMS and poultry waste unfolds an opportunity to tackle waste management challenges while concurrently reaping the rewards of environmental enhancement through resource recycling and the generation of renewable energy [59,60,61]. This is especially pertinent considering that these lignocellulosic agro-wastes represent accessible raw biomass that can be harnessed to produce biofuels and bioenergy. The process is thoughtfully elucidated in Figure 6.

Recent studies have made significant strides in exploring the myriad possibilities presented by spent mushroom substrate (SMS) and poultry waste within the realm of agricultural practices, effectively highlighting the intrinsic value of these resources as versatile organic assets.

In one notable endeavor, Zhang et al. [59] delved into the realm of co-composting, evaluating the potential of chicken manure (CM) in conjunction with SMS, tobacco powder, and vinasse/mushroom bran. This study placed a strong emphasis on harnessing SMS and poultry waste to elevate compost quality while simultaneously mitigating greenhouse gas emissions.

Expanding on this theme, another study by Zhang et al. [60] ventured into the benefits reaped from the combined utilization of SMS and layer manure in co-composting, with a specific focus on the poultry industry. Notably, the incorporation of bamboo biochar into this process emerged as a strategic enhancement, significantly improving nitrogen preservation, and mitigating nutrient loss. This pioneering research underscores the remarkable potential of SMS and poultry waste in optimizing nutrient management practices within poultry farming.

In a different research domain, Gao et al. [61] took on the challenge of anaerobic co-digestion, where SMS was coupled with various forms of livestock manure, including poultry waste, for the purpose of biogas production. This study brought to light the substantial enhancement in biogas production achieved through the co-digestion of SMS and poultry waste, effectively spotlighting the potential of these organic resources as drivers of renewable energy generation within the poultry industry.

Turning to the domain of energy recovery, Shu et al. [62] undertook a study focused on the two-stage anaerobic digestion of SMS and CM, with the aim of optimizing methane production. The outcomes demonstrated a notable increase in methane production, showcasing the substantial potential of SMS and poultry waste in facilitating energy recovery within poultry farming operations.

Furthermore, Nyinoh and Utume [37] explored the vital realm of bioremediation, concentrating their efforts on the remediation of oil-contaminated soil employing Pleurotus ostreatus spent substrate. This study underscored the potential of SMS and poultry waste in addressing environmental contamination issues, including those entwined with poultry farming.

In collective harmony, these studies underscore the versatile potential of SMS and poultry waste across a spectrum of agricultural applications, including biogas production, precision nutrient management, soil enhancement, and bioremediation, with a specific focus on the dynamic context of the poultry industry. Leveraging these organic resources can significantly contribute to the promotion of sustainable agricultural practices and the efficient management of waste in poultry farming, yielding both ecological and operational benefits.

#### 4.1.3. Animal Nutrition

**Figure 7:**
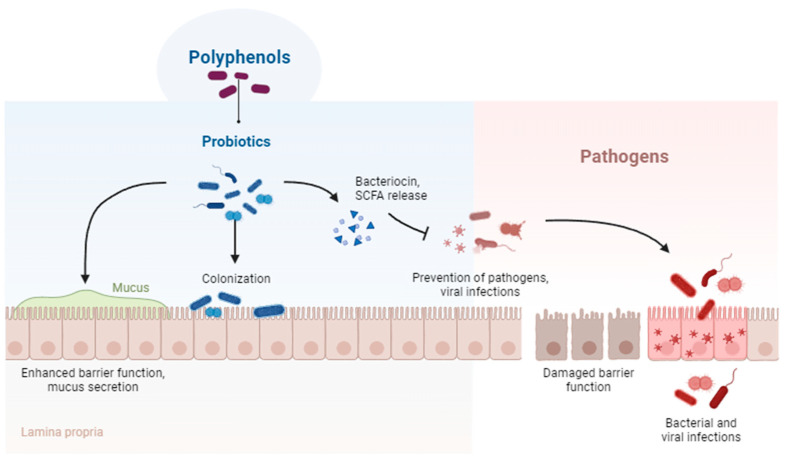
Metabolism of (poly)phenols in the colon and absorption of the metabolites [63].

Spent mushroom substrate holds promising potential as a valuable ingredient in animal diets. SMS, derived from the cultivation of mushrooms, offers a rich source of nutrients and bioactive compounds that can benefit livestock nutrition and health. Studies have demonstrated the effectiveness of incorporating SMS in animal feed formulations. Notably, compounds like ellagitannins, lignans, isoflavones, and flavanones, which play a role in gut microbiota, are likely to provide health benefits in the gastro-intestinal tract. Additionally, the metabolites produced by gut microbiota from these compounds might contribute to the broader systemic effects associated with their parent compounds, as observed in Figure 7 [63].

However, the evidence of the biological activity and the mechanisms underlying the effects of these metabolites in vivo is still unclear, meaning incorporating SMS in poultry diets needs more studies, meaning fewer studies were found. One such study by Noopan et al. [64] explored the impact of using SMS derived from *Cordyceps militaris* on broilers’ growth performance and blood metabolite levels. The researchers found that incorporating SMS into the broilers’ diet positively influenced their growth performance, indicating its potential as a beneficial feed supplement. In another study by Chuang et al. [65], waste mushroom compost was evaluated as a broiler feed supplement. The researchers assessed its effects on broilers’ fat metabolism and antioxidant capacity. The results demonstrated that incorporating waste mushroom compost into the diet had favorable effects on fat metabolism and enhanced the antioxidant capacity of the broilers. This suggests that SMS can serve as a valuable feed supplement for promoting better health and metabolic function in broilers.

Foluke et al. [66] investigated the feasibility of utilizing SMS as a replacement for wheat bran in the diet of broilers. Their study aimed to determine the potential of SMS as an alternative dietary ingredient that could provide similar or improved nutritional benefits for broilers. The findings revealed that SMS could be a suitable replacement for wheat bran in the broilers’ diet, indicating its versatility and potential as a feed ingredient. In a study by Azevedo et al. [67], the researchers explored the use of spent substrate derived from *Pleurotus sajor-caju* in the diet of broiler chickens. They examined its effects on the performance of the chickens, focusing on various parameters. The results showed that incorporating the spent substrate into the diet of broiler chickens positively influenced their performance, suggesting that SMS could be a valuable component for optimizing broiler growth and development.

Lastly, Machado et al. [68] focused on using spent mushroom substrate derived from *Agaricus blazei* in the diet of broiler chicks. Their study aimed to determine the potential benefits of incorporating this specific SMS into the diet of young broilers. The researchers found that the inclusion of *Agaricus blazei* spent substrate positively affected the growth and development of broiler chicks, further supporting the potential of SMS as a valuable dietary component for poultry.

These studies highlight the promising effects of utilizing spent mushroom substrate (SMS) in poultry diets, specifically broilers, chickens, and chicks. The incorporation of SMS as a feed supplement has been shown to positively impact growth performance, blood metabolite levels, fat metabolism, antioxidant capacity, and overall performance in these poultry species. These findings emphasize the potential of SMS as an alternative and beneficial feed ingredient in the poultry industry, offering a sustainable and economically viable approach to enhancing poultry nutrition and productivity. These studies collectively contribute to a body of evidence that highlights the beneficial effects of SMS on animal health and performance. While each study provides insights into specific aspects of animal nutrition, their consistency in observing positive outcomes strengthens the overall conclusions drawn. Furthermore, the safety implications are indirectly supported by the absence of reported negative effects in these studies, However, more research is needed into the matter due to the lack of studies.

#### 4.1.4. Summary of the Selected Studies

In this review article, we compile a comprehensive table summarizing the pivotal research studies cited in this article. This table functions as an invaluable resource, offering concise insights into the various research topics investigated by authors and the primary findings or conclusions they have drawn from their research endeavors. By presenting this carefully curated selection of studies, our aim is to provide a holistic overview of the current state of knowledge at the intersection of spent mushroom substrate and poultry production, highlighting the innovative solutions, challenges, and prospects that have arisen from these investigations. A summary of the selected studies can be found in Table 1. 

### 4.2. Notes on the Excluded Research

Some studies appeared to meet the inclusion criteria but were excluded when better analyzed. The main criterion to exclude research was using spent mushroom and poultry products, such as poultry manure, separately, not showing synergetic results. 

Gagnon et al. [69] explore the contribution of on-farm and industrial composts to soil pH and the enrichment of available nutrients and metals. It investigates the effects of different compost types on soil properties and nutrient availability, providing insights into compost application’s potential benefits and limitations. At the same time, Nicol and Burlakoti [70] examine the effect of aerobic compost tea inputs and application methods on protecting tomato plants from *Phytophthora capsici*, a destructive plant pathogen. The study investigates the efficacy of compost tea in disease management, highlighting its potential as a sustainable alternative to chemical fungicides.

While these studies may not specifically address the synergistic use of spent mushroom substrate (SMS) and poultry manure, they still provide valuable insights into the use of composts in agriculture. Although they do not meet the selection criteria for this paper, they can be valuable resources for future research on composting and nutrient management in agricultural systems.

It is important to note that excluding these articles does not diminish the significance of the studies in the discussion. The selected studies specifically focused on the combined use of SMS and poultry manure and demonstrated their synergistic effects as fertilizers and in reducing gas emissions.

### 4.3. Future Research

As the poultry industry seeks sustainable and environmentally friendly solutions, the utilization of spent mushroom substrate (SMS) emerges as a promising alternative. This section aims to present a more structured approach, addressing key aspects of SMS utilization in poultry production to bridge existing gaps and advance our understanding. Firstly, an elucidation of SMS’s composition, bioactive contents, and biological properties is essential. Furthermore, exploring various processing methods for SMS becomes crucial, but it is equally important to acknowledge the limitations associated with these techniques. To address these limitations, future research should focus on innovative processing approaches like enzymatic treatments, fermentation, or extrusion processes, aiming to enhance the nutritional value and bioavailability of SMS.

Delving into the challenges of SMS incorporation in animal nutrition, specifically as a prebiotic, requires a comprehensive approach. Researchers should conduct in-depth investigations into SMS’s impact on the modulation of poultry gut microbiota. Understanding the underlying mechanisms of how SMS promotes beneficial gut bacteria, possibly by stimulating short-chain fatty acid production or enhancing mucin production, is pivotal. Beyond this, exploring SMS’s effects on gut health parameters, including intestinal morphology, barrier function, and immune response, should be a priority.

A critical point of focus is identifying and quantifying specific bioactive compounds within SMS, such as polysaccharides, phenolic compounds, and antioxidants. Future research should concentrate on elucidating the physiological effects of these compounds on poultry health, including their impact on antioxidant capacity, immune modulation, and disease resistance. Moreover, considering the synergistic effects of combining SMS with other feed additives or functional ingredients could lead to enhanced nutraceutical properties in poultry diets.

To address environmental concerns, SMS’s potential role in poultry manure management requires exploration. Research should investigate SMS’s effectiveness as a bedding material in poultry housing to enhance manure handling and minimize ammonia emissions. Additionally, assessing the influence of SMS incorporation on reducing greenhouse gas emissions, nutrient composition, and the stability of compost or fertilizer derived from poultry manure-SMS blends is pivotal for sustainable farming practices.

To achieve optimal utilization of SMS in poultry production, innovative processing techniques should be at the forefront. These could include enzymatic treatments, fermentation, or extrusion processes to maximize SMS’s nutritional value. Developing balanced feed formulations that efficiently incorporate SMS while ensuring optimal growth, performance, and cost-efficiency in poultry production is a key consideration.

Advanced analytical techniques like metabolomics or genomics should be harnessed to unravel complex interactions between SMS components and poultry physiology. By embracing a holistic and interdisciplinary approach, researchers can pave the way for a thorough understanding of SMS’s potential in poultry production.

The future holds significant promise for utilizing spent mushroom substrate in poultry production. By systematically addressing the outlined research directions—ranging from prebiotic exploration to waste management—we can harness the full potential of SMS to enhance poultry health, bolster waste management practices, and foster sustainable production systems. Collaborative efforts between researchers, industry stakeholders, and policymakers will be pivotal in realizing these advancements.

## 5. Conclusions

In conclusion, SMS stands as a valuable resource with myriad environmentally sustainable applications. As a biofertilizer and soil enhancer, SMS offers a cost-effective, nutrient-rich alternative to chemical fertilizers, resulting in improved soil health and a diminished ecological footprint. The comprehensive examination of studies within this manuscript consistently demonstrated the positive impacts of SMS-based composts, including increased levels of soil organic carbon (C) and nitrogen (N) fractions, enhanced soil microbial growth and activity, and an overall improvement in soil fertility.

Furthermore, SMS exhibits versatility by serving as a key player in renewable energy production, utilizing agro-industrial biomass and thereby reducing our dependence on non-renewable energy sources. In the realm of environmental remediation, SMS proves its worth as a bioremediation agent, facilitating the removal and degradation of contaminants in air, soil, and water. Notably, co-composting SMS with poultry manure has demonstrated the potential to mitigate greenhouse gas emissions, particularly ammonia (NH_3_) and nitrogen oxide (N_2_O) volatilization.

Moreover, SMS has garnered attention as a promising ingredient in poultry nutrition due to its high fiber content, protein content, and bioactive compounds. These attributes have been shown to enhance poultry growth, health, and overall performance. By harnessing the nutrient-rich properties of SMS by-products, the poultry industry can simultaneously reduce its reliance on chemical fertilizers, promote soil health, and contribute to the overall sustainability of the sector.

As we look forward, continued research and development in these areas will undoubtedly play a pivotal role in advancing sustainable agriculture, bioenergy production, environmental remediation, and improvements in animal nutrition. This collective effort will lead us closer to a more environmentally responsible and resilient future.

## Figures and Tables

**Table 1:** Summary of the selected studies, the effects, and corresponding references, according to the field of study (agriculture and animal nutrition).

Chapter	Effects	Reference
Alternatives to Conventional Fertilizers	Composted animal manures as alternative to inorganic fertilizers in intensive vegetable production systems.	[43,44]
	Effects of SMS and poultry manure compost on honeydew melon seedlings and production.	[47,48]
	Onion cultivars’ growth and yield improvement with SMS application.	[49]
	Cauliflower growth and quality enhancement with SMS application.	[50]
	Utilization of pelleted organo-mineral fertilizers derived from composted waste for grasslands.	[51]
	Impact of composted manure and chemical fertilizers, including SMS, on apple orchard soil.	[52]
	Composted media derived from waxed corrugated cardboard with SMS as soil amendment and growing medium for woody ornamentals.	[53,54]
	Biochemical and microbiological properties of co-composting SMS and chicken feathers.	[55]
	Co-composting of SMS, carnation wastes, CM, and cattle manure.	[56]
	Co-composting SMS and CM with garden waste addition.	[57]
	Changes in microbial community during co-composting of swine and poultry manure with SMS.	[58]
	Effects of SMS and poultry waste on composting and soil improvement.	[45,46]
Renewable Energy and Gas Emissions	Co-composting of CM with SMS to enhance compost quality and reduce greenhouse gas emissions.	[59]
	Benefits of using SMS and layer manure in co-composting with bamboo biochar for nitrogen conservation.	[60]
	Anaerobic co-digestion of SMS and poultry waste for biogas production.	[61]
	Two-stage anaerobic digestion of SMS and CM to enhance methane production.	[62]
	Bioremediation of oil-contaminated soil using *Pleurotus ostreatus* spent substrate.	[37]
Animal nutrition	Impact of SMS derived from *Cordyceps militaris* on growth performance and blood metabolite levels of broilers.	[64]
	Evaluation of waste mushroom compost as a feed supplement for broilers.	[65]
	Feasibility of utilizing SMS as a replacement for wheat bran in the diet of broilers.	[66]
	Use of spent substrate derived from *Pleurotus sajor-caju* in the diet of broiler chickens.	[67]
	Effects of spent mushroom substrate derived from *Agaricus blazei* in the diet of broiler chicks.	[68]
